# Pavlovian Fear Conditioning Activates a Common Pattern of Neurons in the Lateral Amygdala of Individual Brains

**DOI:** 10.1371/journal.pone.0015698

**Published:** 2011-01-12

**Authors:** Hadley C. Bergstrom, Craig G. McDonald, Luke R. Johnson

**Affiliations:** 1 Psychiatry and Neuroscience, School of Medicine, Uniformed Services University (USU), Bethesda, Maryland, United States of America; 2 Center for Neuroscience and Regenerative Medicine (CNRM), Rockville, Maryland, United States of America; 3 Psychology, George Mason University, Fairfax, Virginia, United States of America; 4 Center for the Study of Traumatic Stress (CSTS), Bethesda, Maryland, United States of America; VU University, The Netherlands

## Abstract

Understanding the physical encoding of a memory (the engram) is a fundamental question in neuroscience. Although it has been established that the lateral amygdala is a key site for encoding associative fear memory, it is currently unclear whether the spatial distribution of neurons encoding a given memory is random or stable. Here we used spatial principal components analysis to quantify the topography of activated neurons, in a select region of the lateral amygdala, from rat brains encoding a Pavlovian conditioned fear memory. Our results demonstrate a stable, spatially patterned organization of amygdala neurons are activated during the formation of a Pavlovian conditioned fear memory. We suggest that this stable neuronal assembly constitutes a spatial dimension of the engram.

## Introduction

Understanding the physical encoding of a memory (the engram) in neuronal networks is a fundamental challenge for neuroscience [1], [2], [3]. A key question to be addressed about the engram is what principle underlies the organization of neurons storing a memory. Pavlovian fear conditioning is a form of associative memory formation where a conditioned stimulus (CS) such as an auditory tone is paired with a fear arousing unconditioned stimulus (US) such as a foot shock. As a result a memory is formed which allows the CS to elicit freezing, a behavioral index of fear. Synaptic plasticity in the lateral amygdala (LA) is critical to the establishment of this memory [4], [5], [6], [7], [8]. Plasticity of synaptic strength and neuronal structure is dependent upon phosphorylation of extracellular signal-regulated kinase, a mitogen-activated protein kinase (ERK/MAPK) [9], [10]. Pavlovian fear conditioning is dependent upon phosphorylation of ERK/MAPK (pMAPK) in the LA, which is detectable as both an increase in pMAPK protein, and pMAPK expressing neurons [11], [12].

Within a memory-storing nucleus, such as the lateral amygdala (LA), it is not understood if the distribution of neurons encoding a given memory is random or spatially organized. In this study, we asked whether Pavlovian auditory fear conditioning in intact animals is associated with a unique topography of pMAPK labeled neurons in the LA and whether this pattern is consistent across animals storing the same fear memory.

To visualize the distribution of pMAPK activated neurons we generated density heat maps at an anatomically matched region of the LA. Next, we applied spatial principal components analysis (sPCA) to quantify the spatial distribution reflected by the density heat maps. sPCA is a data reduction technique used to capture patterns of covariance from large datasets [13], [14], [15], [16], [17]. In this study we used sPCA to extract a spatial pattern of activated LA neurons that could statistically distinguish between brains that did or did not acquire an auditory fear memory.

We found a unique pattern of neuronal activation in the LA that was associated with the formation of an auditory fear memory. The topography was consistent across brains encoding the same fear memory, suggesting that the spatial distribution of LA neurons associated with fear memory encoding is stable.

## Results

### Section alignment

**Figure 1:**
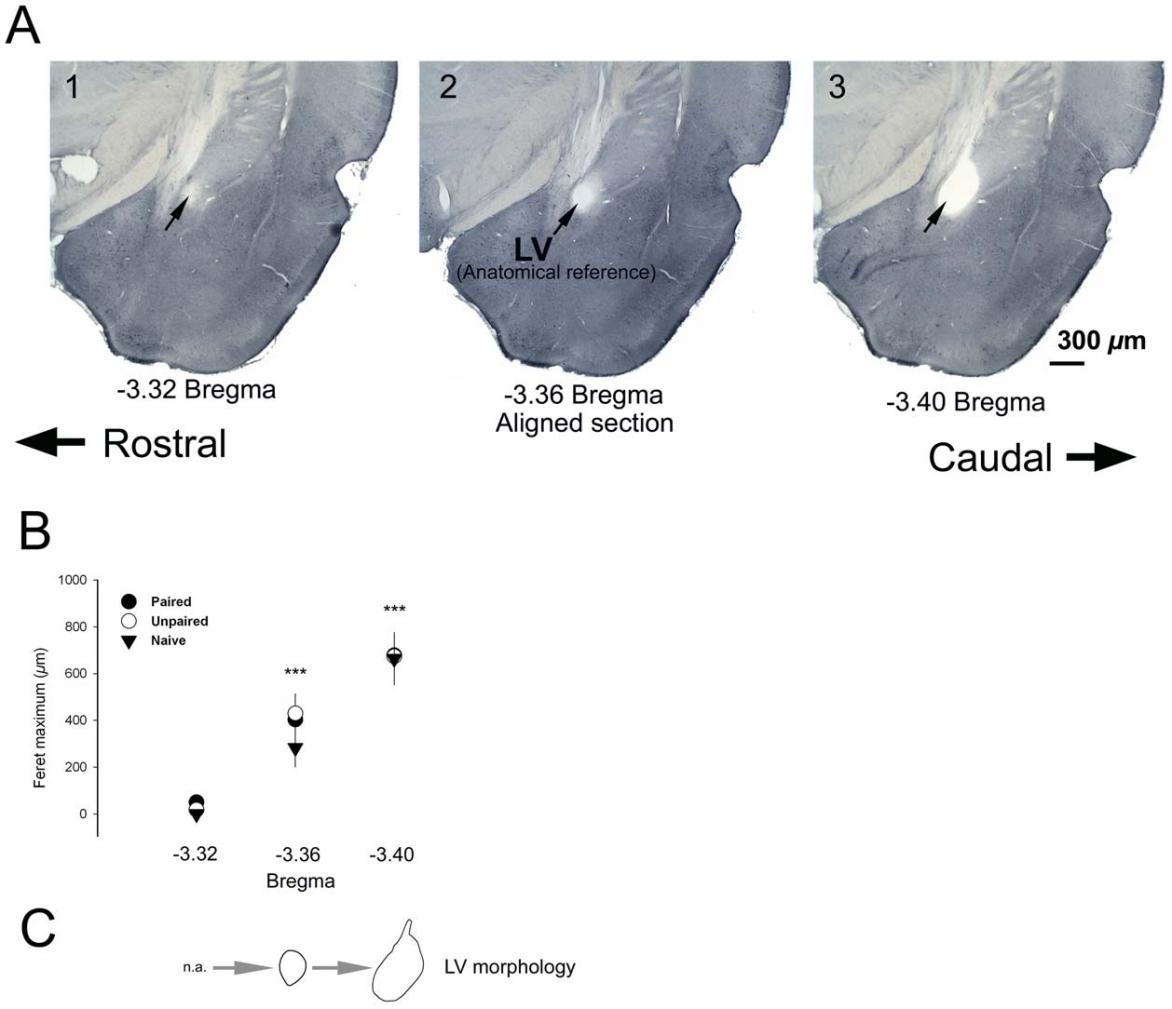
The entrance of the lateral ventricle was used as an anatomical landmark for section alignment. (A) Representative photomicrographs (2x magnification) of three consecutive 40 *µ*m sections depicting (1) the “blind pouch” (indicated by arrowhead) rostral to the initial opening of the lateral ventricle (LV), (2) initial opening of the LV (Bregma −3.36) and (3) the LV (indicated by arrowhead) caudal to the initial opening of the LV (B) The maximum feret measurement of the LV was significantly different at the section rostral (Bregma −3.32) and caudal (Bregma −3.40) to the section chosen for mapping (Bregma −3.36). (C) Representative reconstructions of the morphology of the LV at Bregma −3.32, −3.36 and −3.40. The reconstructions of the LV for consecutive sections are representative of the mean maximum feret distance for all subjects. Circles represent the mean LV feret measurement ± standard error of the mean. *** indicates *p*<.00001.

We set out to provide a quantitative measure of coronal brain section alignment, rather than a qualitative measure as is traditionally used for comparing brain sections. We identified the lateral ventricle as a structure that could both be accurately measured and importantly, it showed rapid change from section to section which allows brain sections to be quantitatively assigned to sequential groups. In order to verify the alignment of the section across subjects, the contour of the entrance to the LV was digitally reconstructed and the maximum feret length was statistically compared (ANOVA) between conditions. The maximum feret length is the longest distance of the contour as if a caliper was used to make the measurement across the two opposing sides (NeuroExplorer, MBF Bioscience, VT). To verify that the section chosen for mapping was significantly different from adjacent sections, a paired *t*-test was used to compare the maximum feret measurement of the LV across three consecutive sections (Figure 1). To assess the degree of similarity between subjects for the section chosen for pMAPK neuron mapping, z-scores were computed from each maximum feret measurement of the lateral ventricle (LV) and outliers were determined. Z-scores greater than 3.29 were considered outliers. No z-scores exceeded the predetermined cut-off of 3.29 so it can be assumed that each section was matched across subjects. The maximum feret measurement of the LV was also compared between experimental conditions to rule out whether artifactual differences due to misalignment of sections may have contributed to the observed between-group differences. There was not a significant difference (*p* = .49) in the maximum feret distance between P5 (403.5±100.5), UP5 (430.0±83.5) and N (283.2±82.4) groups, indicating that the section chosen for mapping was closely aligned between experimental conditions. The maximum feret measurement of the LV for the section chosen for mapping (Bregma −3.36) was significantly different from the section rostral (−3.32; *p* = .000003) and caudal (−3.40; *p* = .000008), confirming the initial opening of the LV as a useful anatomical reference for section alignment between subjects. Together, these results confirm that differences in pMAPK neuron distribution between experimental conditions are not due to misalignment of sections between groups (Figure 1).

### pMAPK neuron density

**Figure 2:**
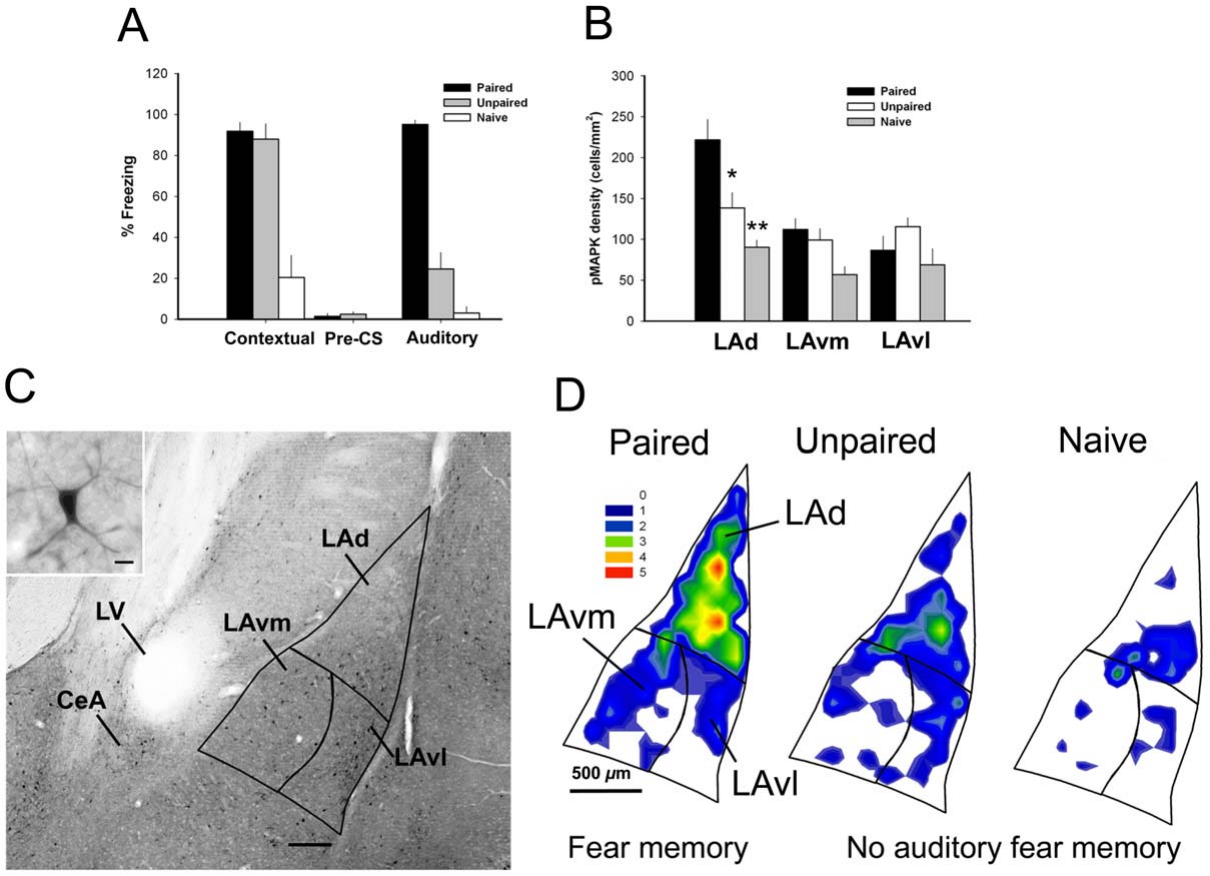
Auditory fear conditioning was accompanied by greater density of pMAPK expressing neurons in the LAd. (A) Paired presentation of the tone and shock produced greater freezing levels when the auditory CS was presented alone in a novel environment relative to the explicitly Unpaired and Naïve control conditions (one-way ANOVA; Bonferroni post-hoc). Freezing prior to the presentation of the auditory CS in a novel chamber (pre-CS) was low for all conditions (B) pMAPK neuron density was increased in the P5 relative to both control conditions for the LaD (Multivariate ANOVA with Bonferroni post-hoc). (C) Photomicrograph of a pMAPK labeled section of LA subnuclei (4X magnification; scale bar  = 100 *µ*m) and representative pMAPK labeled neuron from the LAd (40X magnification; scale bar  = 10 *µ*m). (D) Micro density heat maps of the LAd, LAvm and LAvl sub regions of the LA depicting the distribution of pMAPK labeled cells in the Paired, Unpaired and Naïve conditions. The colors for each bin reflect an estimation of spatial density from low (blue) to high (red).* denotes *p*<.05, ** *p*<.01, *** *p*<.001.

Using MANOVA, we compared the relative change in pMAPK neuron density among LA subnuclei, which included the dorsal (LAd), ventromedial (LAvm) and ventrolateral (LAvl) subnuclei. Significant differences between conditions were restricted to the LAd (F_2,10_ = 11.5; *p* = .003) (see Results S1 and Figure 2). Apparent increases in pMAPK density were also observed in amygdala regions ventral to the LAd, including the LAvm and LAvl. In the current study of a single matched section these increases were not significantly different. In addition, no differences in pMAPK neuron density among the experimental conditions were found for the MePD (Figure S1). Next we compared the density of pMAPK activated neurons in the LAd from the P5 group to the total number of principal neurons as revealed by calcium/calmodulin-dependent protein kinase II (CaMKII) immunocytochemistry. pMAPK neurons represented 20% (79.8±9.0: 403.0±11.3) of the total principal neurons (pMAPK n = 4; CaMKII n = 4)(Figure S2). This proportion of neurons in the amygdala, activated as a result of associative fear learning, is directly comparable to the proportion of principal neurons identified using different methods to visualize neurons undergoing plasticity, including pCREB [18] and AMPA receptors [19]. Collectively, these data show that association of the tone and shock alters pMAPK neuronal density in the LAd [11], [12]. Consequently we restricted subsequent neuronal topographical analyses of pMAPK expressing neurons to LAd.

### Spatial Principal Components Analysis

**Figure 3:**
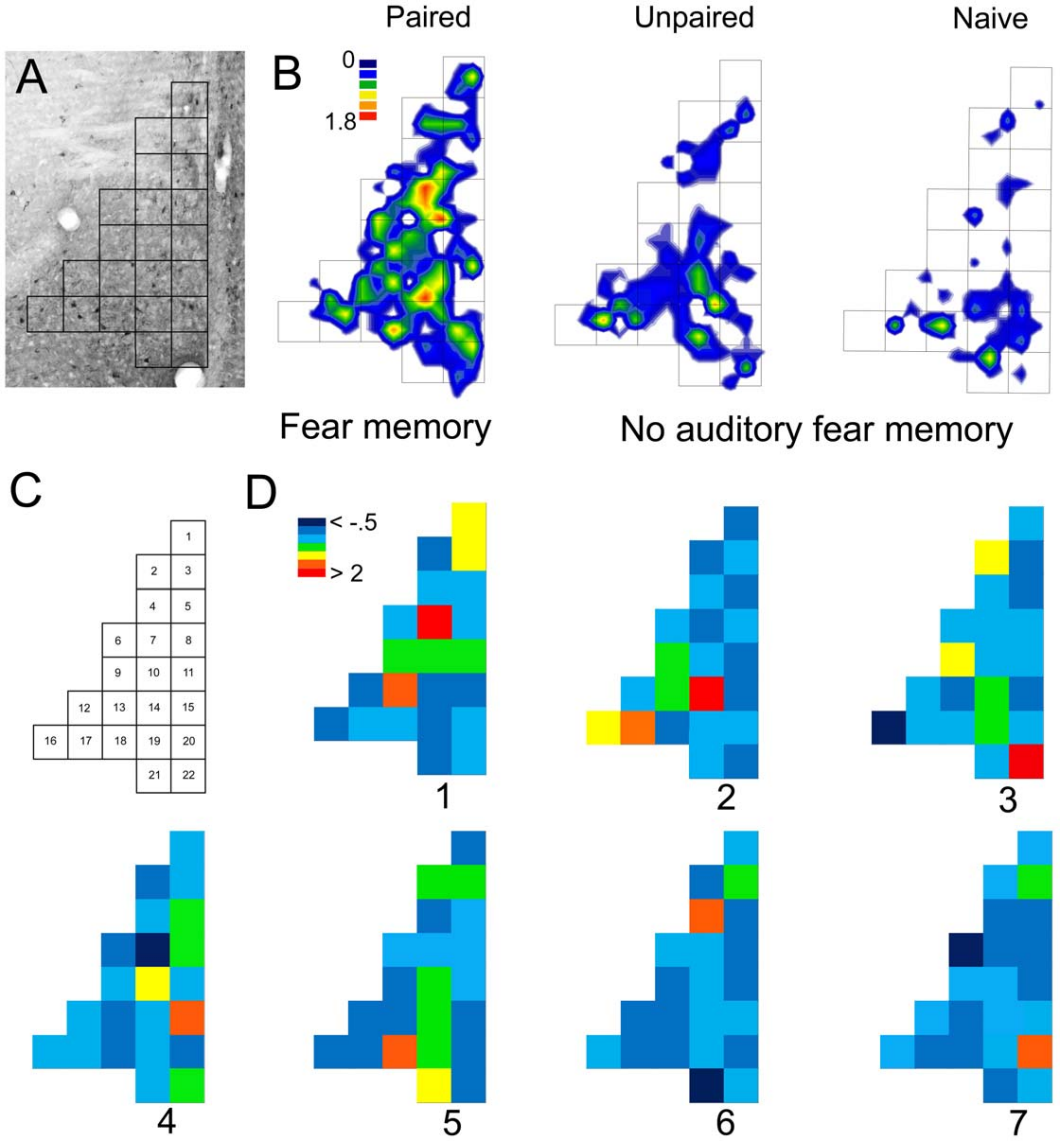
Spatial principal components analysis of the distribution of pMAPK labeling in the LAd. (A) Photomicrograph (4x magnification) of the LaD with grid overlay. (B) Grid showing the numerical layout of bins (120 *µ*m^2^) for the LAd. (C) Micro density maps with grid overlay illustrating the mean distribution of pMAPK labeled neurons for the Paired (auditory fear memory), Unpaired and Naïve (no auditory fear memory) experimental conditions. (D) Component loading maps illustrating the spatial distribution of loading values for each component. The loading values can be interpreted as representing regions of variance in pMAPK activity. The loading maps are ordered according to decreasing eigenvalues with the largest eigenvalue associated with component 1. The component loading maps were generated by first categorizing the frequency of loading values for all 7 factors into bins. Each bin was color coded to reflect incremental loading values from low (dark blue) to high (red).

**Figure 4:**
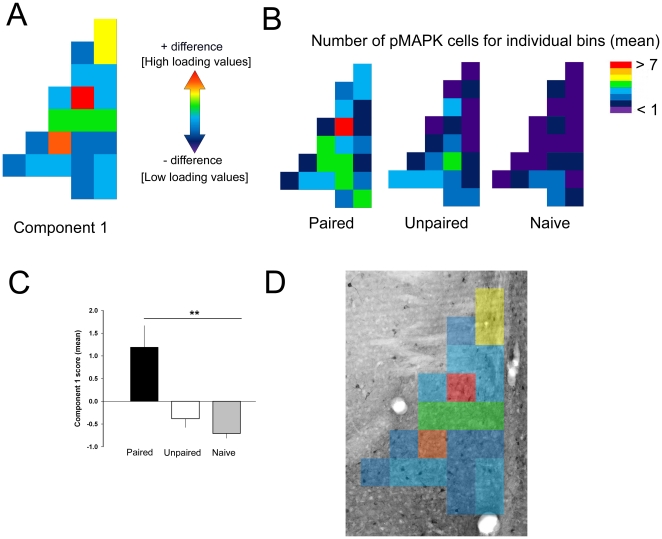
Spatial principal component analysis revealed a pattern of pMAPK activated neurons unique to auditory fear conditioning. (A) Component 1 loading map best represents the difference in pMAPK activity between groups. The loading values can be interpreted as representing regions of difference in pMAPK activity between the experimental conditions as depicted in panel B. (B) Grid maps depicting the mean value of pMAPK activity for each experimental condition. The mean values for pMAPK activated cells were assigned a color value from violet (low cell number) to red (high cell number) (C) The bar graph reflects the mean difference in the component 1 scores between Paired, Unpaired and Naive groups (one-way ANOVA; Bonferroni post-hoc). (D) Photomicrograph (4 x magnification) of the LAd with superimposed component 1 loading values. ** denotes *p*<0.01.

Density heat maps plotting mean pMAPK neuron density in LAd (Figure 3) revealed a distinct pattern in the distribution of pMAPK neurons in the P5 group compared to controls. These maps suggest that neurons expressing pMAPK due to the association of tone and shock, and thus encoding the engram, may be topographically organized in the LAd. In order to evaluate the topographical stability of pMAPK expressing neurons across individuals and experimental groups, we performed spatial principal components analysis (sPCA) on neuronal density maps from LAd. sPCA was used to reduce the complex spatial distribution of pMAPK labeled neurons into a less complex set of uncorrelated pattern components (see Results S1, Figure S3). The pattern of labeling is illustrated by the loading values associated with a particular component, and pattern prominence is indicated by the component score for each individual. The analysis revealed a single component with a consistent pattern of pMAPK neuron labeling that was specific to the P5 group (Figure 3 and Figure 4; see Results S1). This pattern represents a stable topography of pMAPK labeling that is associated with the formation of the auditory fear memory (Figure 4).

What appeared to distinguish the distribution of pMAPK labeled neurons in the present experiment was differential labeling in discrete regions within LAd between conditions, an observation consistent with higher loading values (for component 1) in a small proportion of the bins. Significantly higher component scores in the P5 group relative to control groups provided quantitative confirmation of this observation (Figure 4). We also statistically compared the spatial patterns of pMAPK labeled neurons associated with the remaining components (2–7) extracted by the sPCA (See Results S1 and Figure 3). Importantly, we found no differences in factor score with respect to experimental condition for these components. This provides further verification that the pattern of pMAPK labeled neurons reflected by component 1 was unique to the formation of the associative fear memory. Overall, the sPCA showed that the distinguishing feature of the pMAPK topography in the P5 group – as compared to the control groups – was a higher proportion of labeled neurons in discrete areas within the dorsal LAd.

In the P5 group, the greatest number of pMAPK labeled cells (7.0±1.2) was localized within bin 7 (from Bregma: 3.6 mm caudal; 5.65 mm medial; 7.23 mm ventral, Figure 3) [20]. Component 1 loaded highly in this area, and this area possessed significantly more pMAPK neurons in P5 relative to both control groups when compared with an appropriately corrected post hoc test (α/22, *p*<0.002; see Results S1). Additionally, there was a significant difference for bin 13, which was also the location of a high component 1 loading value. The fact that bins with the highest loading values were also the sites of the greatest difference in neuronal number between experimental conditions verifies the use of sPCA in extracting meaningful patterns of variance associated with the experimental manipulation. This finding suggests the possibility that an extremely small portion of the LAd makes a significant contribution to the encoding of Pavlovian auditory fear memory.

## Discussion

The present data provide the first evidence that formation of a Pavlovian fear memory is associated with a unique neural topography in the amygdala. The consistency of the spatial pattern across animals that encoded the same fear memory indicates that the topography of pMAPK neuron activity observed in the present data-set was reliable and non-random.

The ERK/MAPK signaling cascade regulates cyclic-AMP response element binding protein (CREB), a transcription factor regulating protein synthesis underlying memory [18], [21]. Recent data shows that neurons expressing experimentally induced up-regulated CREB are preferentially recruited into the network of LA neurons encoding a Pavlovian fear memory [18], [22]. These data suggest that the inclusion of a given LA neuron into the network is not random but rather depends on the level of CREB expressed in the neuron at the time of the memory formation [18], [21]. The present finding of a stable spatial map of neurons associated with Pavlovian fear conditioning provides evidence for non-random participation of neurons encoding a fear memory.

sPCA is a particularly useful data-driven statistical tool for decoding complex interactions of spatially distributed biological activity. The advantage of using sPCA to a more traditional test of independent samples (e.g., a *t*-test) is that sPCA reduces the large numbers of variables into a smaller set of uncorrelated variables. A comparison of the new, smaller number of variables between experimental conditions significantly reduces familywise error rates resulting from multiple comparisons. Thus, with sPCA it was possible to identify the most common overall pattern of neuron distribution associated with fear memory formation across different animals (Figure 4). In addition, within this pattern it was possible to identify regions of greatest difference (Figure 4).

One potential caveat of the present study was the relatively small number of observations in relation to the number of variables (observation to variable ratio,1∶1.69). Nevertheless, only the first of the seven components extracted by the sPCA reflected the pattern suggested by the density heat maps (Figure 3) and distinguished among experimental conditions (Figure 4). A subsequent ANOVA of group means within each bin provided confirmation that only component 1 reflected a meaningful pattern of variance. These results collectively indicate that the number of observations was sufficient to permit reducing heat maps of high dimensionality into simplified spatial components. Moreover, using the approach developed here future studies may be able to also determine neuronal patterns encoding different sensory CS's and the contextual component of Pavlovian fear memories.

Recent human fMRI [23], [24] and rat [25] data demonstrate a stable topography of activation for specific memories in the hippocampus. Our current data show the principle of a stable topography at the neuron level in the amygdala. Both the finding of a stable neural assembly associated with fear memory formation as well as the use of the spatial principal components analysis method to identify patterns is novel. In addition, these data provide a practical micro map. This map will enable the better isolation and study of important aspects of neuronal plasticity and associative memory in the LA and its subnuclei.

Overall, these data provide the first evidence for a unique neural topography associated with memory formation. Thus, in addition to showing that associative memory encoding is linked to increased numbers of pMAPK activated neurons in LAd, we provide evidence that the engram also has a spatial dimension. A stable, spatially organized neural assembly may be a fundamental feature of the engram by which associative fear memory is encoded.

## Materials and Methods

### Animals

Adult male Sprague-Dawley rats (Taconic) were group housed (2/cage) and maintained on a 12 hr light/dark cycle with food and water were provided ad libitum. Rats were handled on three consecutive days prior to testing. All procedures were conducted in accordance to the National Institute of Health *Guide for the Care and Use of Experimental Animals* and were approved by the Uniformed Services University Institutional Animal Care and Use Committee (PSY-08-697). Experiments were conducted on two parallel cohorts of three experimental groups that were run simultaneously. Rats (N = 25) were randomly assigned to one of three groups: paired (n = 8), unpaired (n = 10) and naïve (n = 7). Following fear conditioning, rats were subdivided into two groups of study, a behavior (paired n = 4, unpaired n = 5 and naïve n = 3) and anatomy (paired n = 4, unpaired n = 5 and naïve n = 4) group.

### Pavlovian auditory fear conditioning

Rats weighing 260–360 g were habituated to the fear conditioning chamber (context A) for 30 minutes one day prior to conditioning. Context A consisted of a Plexiglas rodent conditioning chamber with a metal grid floor (Coulbourn Instruments, Lehigh Valley, PA), illuminated by a single house light, and enclosed within a sound-attenuating chamber. The conditioning chamber was interfaced to a stimulus controller (Coulbourn Instruments, Lehigh Valley, PA). The chamber was cleaned with a 70% EtOH solution between subjects. On conditioning day, rats were placed in context A and left to explore the chamber for three minutes prior to presentation of stimuli. Rats in the paired (P5) group were exposed to five tones (CS; 5 kHz, 75 dB, 20 s) that co-terminated with a foot shock (US; 1.0 mA, 500 ms). In the control conditions, an unpaired (UP5) group was presented with non-overlapping presentations of the CS and US. The presentation of the stimuli for UP5 was ordered so that there was one occurrence in which a CS followed a CS and US followed a US. In this way the tone was less predictive of the occurrence of a shock. The time between stimulus onset (intertrial interval; ITI) for all groups was 30–90 s (60 s mean). Animals were removed from the chamber 60 s following the final stimulus presentation. A naïve (N) group was exposed to the fear conditioning chamber alone for ten minutes. Rats in the behavior group were placed back into the fear conditioning chamber (context A) 24 hours later for analysis of freezing to the original training context (contextual fear memory). Three days following testing for contextual memory, rats were tested for retention of auditory fear conditioning in a novel context (Context B) to mask environmental cues of the conditioning chamber. Context B consisted of plastic flooring covered with fresh bedding, altered geometry and spatial cues (red and black tape) relative to context A, four additional cue lights illuminated continuously, and 1% ammonium hydroxide solution used as a cleaning agent. On the day of auditory fear testing, rats were placed into context B and presented with 10 auditory CSs alone at the identical frequency and dB level as conditioning. An experimenter blind to the experimental condition of the animals scored freezing behavior, a measure of conditioned fear. Freezing was defined as the absence of all movements except those related to respiration [26]. Freezing was scored during the CS presentation (20 s) from digitized videos. A mean freezing value was calculated from the 10 scored freezing episodes and transformed into a percent freezing by dividing the total time spent freezing by the number of scored freezing episodes and dividing by 100. Mean freezing was the dependent variable.

### Immunocytochemistry

In the second (anatomy) cohort, we identified pMAPK activity in principal neurons following auditory fear conditioning using antibodies against ERK/MAPK p42/44 and then mapped neurons in the right LA and medial posterodorsal amygdala (MePD). The MePD served as a control region not associated with Pavlovian fear conditioning [27] (see Results S1 and Figure S1).

### Tissue preparation

pMAPK expression in the LA has been shown to peak at 60 minutes post-auditory fear conditioning [11]. For this reason rats were anesthetized exactly 60 minutes following auditory fear conditioning. Rats were anesthetized via intraperitoneal (i.p.) injection of a ketamine/xylazine (100 mg/kg, 10 mg/kg) cocktail and transcardially perfused through the ascending aorta with ice cold saline (100 mL) followed by ice cold 4% paraformaldehyde/1% glutaraldehyde/0.1 M phosphate buffer saline (PBS) at pH 7.4 (250 mL). For calcium/calmodulin-dependent protein kinase II (CaMK) immunocytochemistry, glutaraldehyde was not included in the fixative. Brains were removed and stored in the fixative overnight (4°C), then stored in PBS for no more than three days. Sequential coronal brain sections containing the LA at −3.36 Bregma (see below) were prepared on a vibratome at 40 *µ*m. All sections were treated with 1% sodium borohydride prior to immunocytochemistry.

### pMAPK

Sections were first blocked with 1% BSA for 1 hr. Next, sections were incubated in a rabbit polyclonal antibody to phospho-p44/42 MAPK (Thr202/Tyr204, 1∶250 dilution, Cell Signaling Technology, Boston, MA) for 24 h at room temperature. Following washing in PBS, slices for pMAPK immunoreactivity were subsequently incubated with biotinylated goat anti-rabbit IgG (1∶200 dilution, Vector Laboratories, Burlingame, CA) for 30 min. Slices were then washed again in PBS and incubated in avidin-biotin HRP complex (ABC Elite, Vector Laboratories, Burlingame, CA). After a final wash in PBS, activated neurons were visualized using SG chromagen (Vector Laboratories, Burlingame, CA). Sections were mounted in numerical order on gelatin subbed slides and air dried, then dehydrated in a graded series of alcohol, xylene and coverslipped.

### CaMK

Following a blocking step identical to that described above, sections were incubated overnight at room temperature in a mouse monoclonal antibody to the alpha subunit of CaMK (20 *µ*g/mL; clone 6G9; Millipore, Billerica, MA). Slices were rinsed in PBS and then incubated in biotinylated goat anti-mouse IgG (1∶200, Vector Laboratories, Burlingame, CA) for 30 min. Subsequent processing steps were identical to that described above.

### Section alignment

Analysis was restricted to the X and Y dimensions of single 40 *µ*m section. The use of a single section maximized the accuracy of direct comparisons among animals. In order to achieve the highest spatial resolution possible, the section from which pMAPK labeled neurons in the LA were mapped was aligned across subjects using the entrance to the lateral ventricle (LV) as an anatomical marker (Bregma −3.36 mm) (Figure 1). At this level, the dorsal (LAd), ventromedial (LAvm), ventrolateral (LAvl) and medial posterodorsal (MePD) subdivisions of the amygdala complex are well represented (Figure 1).

### Neuronal mapping

For all neuronal mapping the experimenter was blind to experimental condition. For a density measurement of pMAPK labeled neurons for each subregion of the LA, the anatomical boundaries of the LA were first demarcated at 4x magnification using a contour tracing tool (Neurolucida, MBF Biosciences, VT) on an image of the LA from a rat brain atlas [20] at the appropriate Bregma (−3.36 mm). Thus the dimensions of the contour were identical between experimental groups. The (XY) coordinates of individually labeled pMAPK neurons within the LA were marked at 20X magnification. NeuroExplorer (MBF Biosciences, VT) was used to quantify markers (XY coordinates) and contours. The density of pMAPK labeling was calculated as the ratio between the total number of pMAPK labeled neurons and the contour area (mm^2^) of each LA subnucleus. Multivariate ANOVA (MANOVA) was used to detect group difference among LA subnucei.

### Spatial principal components analysis of pMAPK labeled cells

To reduce the complex spatial distribution of pMAPK labeled cells into a more simplified structure, spatial principal components analysis (sPCA) was applied to 698 total neurons from the LAd. The result of sPCA is set of component loadings and scores. Component loadings reflect orthogonal patterns of variance in the distribution of pMAPK neurons. Component scores reflect the contribution of each component or ‘pattern prominence’ in each of the experimental groups. Thus, grouping scores by condition reveals variability associated with experimental manipulation.

We chose to focus our analysis on the LAd because a significant difference in pMAPK labeled neuron density between the P5, UP5 and N conditions was localized to the LAd (Figure 2). No differences between all experimental conditions were found for either the LAvm or LAvl subdivisions (Figure 2). In addition, the LAd receives strong projections from the medial geniculate nucleus of the thalamus (auditory thalamus) [28]. To determine the spatial distribution of pMAPK using sPCA, a virtual grid was constructed and aligned with the anatomical boundaries of the LAd [20]. To construct the grid, the contour of the LAd was partitioned into equal sized sub regions (bins). The bin dimensions were determined by the area of the LAd and the mean number of data points (pMAPK labeled neurons) for all subjects [29]. Bins for the LAd measured 120 *µ*m^2^. Principal neurons in the amygdala are on average 15–20 *µ*m in diameter [30]. The ratio of pMAPK activated neurons to surface area (mm^2^) of the LAd was 1∶80. Therefore, the dimensions of each bin (120 *µ*m^2^) in the LAd allow for an appropriate level of spatial resolution for mapping patterns of pMAPK activated neurons in the LAd. Bins were arranged within the borders of the LAd so as to account for the maximum amount of area (Figure 3). The geometry of the grid was determined by the anatomical shape of the LaD [20]. The total number of pMAPK labeled neurons within each bin was considered the dependent variable in the sPCA. We used a covariance association matrix, treating individual bins as variables. We applied varimax rotation, followed by promax rotation (kappa 2) to obtain simple structure. We also carried out the analysis with a varimax rotation, which provided a virtually identical solution. The similarity in outcome following both approaches illustrates orthogonality among principal components. The loading maps for each component were evaluated with the goal of determining similarity to the spatial distribution of the mean density heat maps for the P5, UP5 and N groups (Figure 3).

The scores for each component were statistically compared using analysis of variance (ANOVA). Subsequent post hoc analysis was performed with a Bonferroni test.

## Supporting Information

Figure S1 **Spatial principal components analysis of the medial amygdala.** There were no differences in overall density or pattern of pMAPK labeled neurons in the MePD between experimental conditions. (A) Diagram of the MePD at Bregma -3.36 and relevant anatomical landmarks [20] (B) Representative photomicrograph of MePD with grid overlay used for sPCA. Bins for the MePD measured 140 *µ*m^2^ (C) There was no difference in the density of pMAPK labeled neurons between experimental conditions. (D) Micro density heat maps depicting the distribution of pMAPK labeling in the MePD for the Paired, Unpaired and Naïve conditions. Bars represent mean pMAPK neuron density ± standard error of the mean.
(TIF)

Figure S2 **Density of CaMK neurons in the LAd.** pMAPK activated neurons from the LAd in the P5 group represented 19.8% of the total number of principal neurons as revealed by calcium/calmodulin-dependent protein kinase II (CaMK) immunocytochemistry. Bars represent mean pMAPK and CaMK density ± standard error of the mean.
(TIF)

Figure S3 **Density heat maps of the LAd for all subjects.** Micro density heat maps of the LAd depicting the distribution of pMAPK labeled cells for all subjects in the Paired, Unpaired and Naïve conditions. To construct the maps, XY coordinates for each pMAPK activated cell were categorized into bin that measured 50 *µ*m^2^. The data points that fell into each bin were counted and placed into a matrix that fit the anatomical dimensions of the LAd (1200 *µ*m^2^). The colors for each bin reflect an estimation of spatial density from low (blue) to high (red).
(TIF)

Results S1 A topography of amygdala neurons.
(DOCX)
