# Supplementary figures and images for: Pavlovian Fear Conditioning Activates a Common Pattern of Neurons in the Lateral Amygdala of Individual Brains
Source: PLoS One. 2011 Jan 12;6(1):e15698. doi: 10.1371/journal.pone.0015698 (PMC3020219; doi:10.1371/journal.pone.0015698)

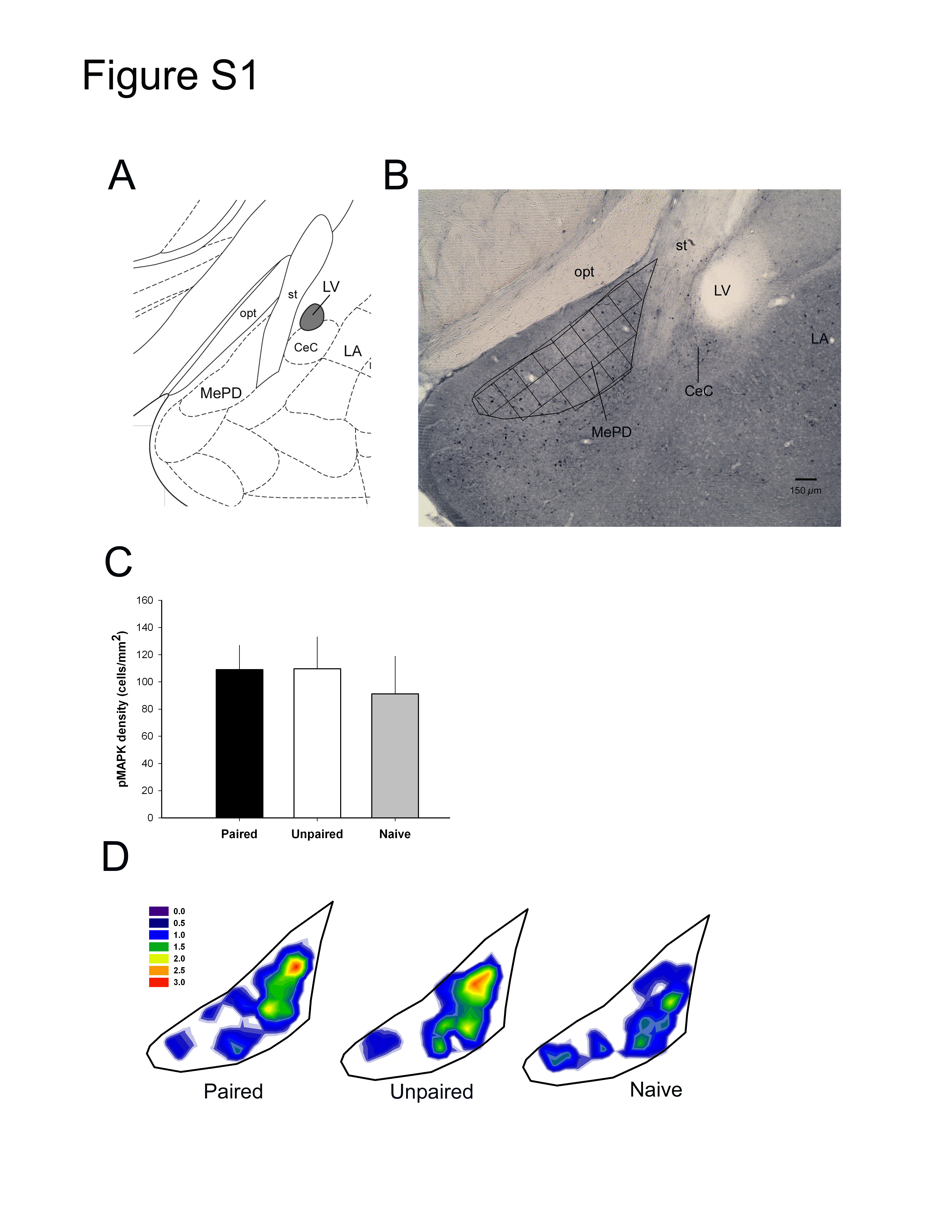

Supplement: Figure S1 — Spatial principal components analysis of the medial amygdala. There were no differences in overall density or pattern of pMAPK labeled neurons in the MePD between experimental conditions. (A) Diagram of the MePD at Bregma -3.36 and relevant anatomical landmarks [20] (B) Representative photomicrograph of MePD with grid overlay used for sPCA. Bins for the MePD measured 140 µm2 (C) There was no difference in the density of pMAPK labeled neurons between experimental conditions. (D) Micro density heat maps depicting the distribution of pMAPK labeling in the MePD for the Paired, Unpaired and Naïve conditions. Bars represent mean pMAPK neuron density ± standard error of the mean. (TIF) [file pone.0015698.s001.tif]

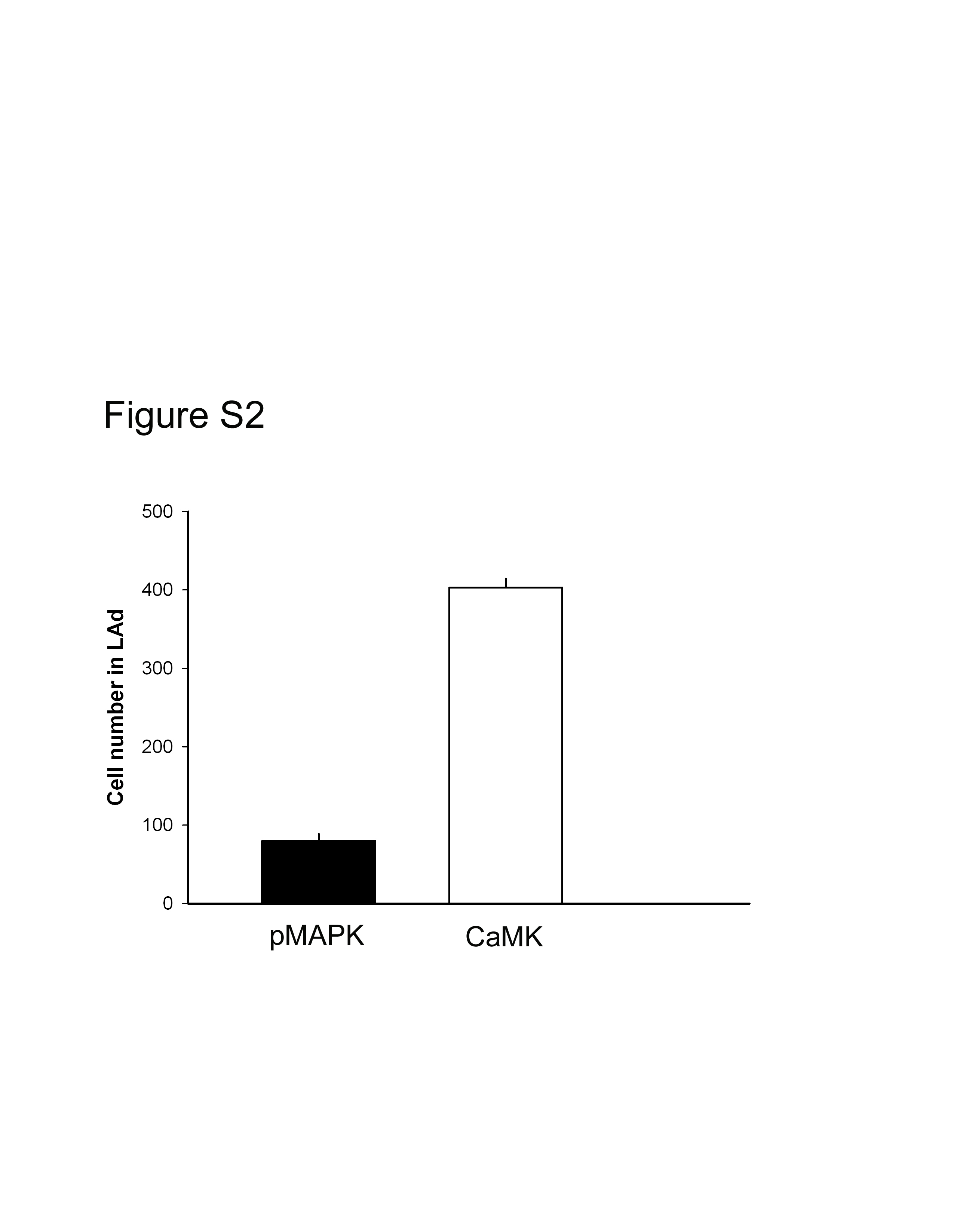

Supplement: Figure S2 — Density of CaMK neurons in the LAd. pMAPK activated neurons from the LAd in the P5 group represented 19.8% of the total number of principal neurons as revealed by calcium/calmodulin-dependent protein kinase II (CaMK) immunocytochemistry. Bars represent mean pMAPK and CaMK density ± standard error of the mean. (TIF) [file pone.0015698.s002.tif]

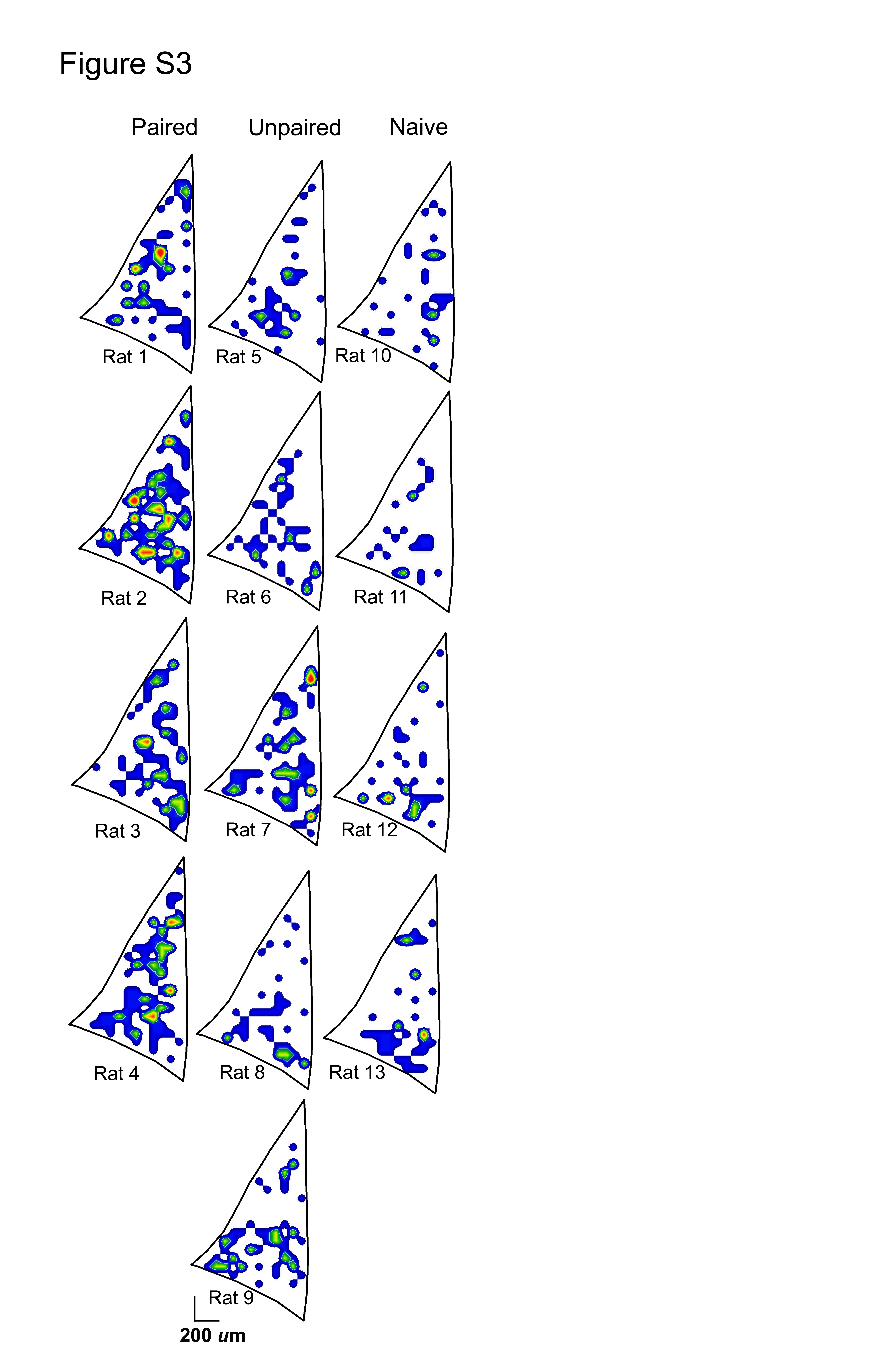

Supplement: Figure S3 — Density heat maps of the LAd for all subjects. Micro density heat maps of the LAd depicting the distribution of pMAPK labeled cells for all subjects in the Paired, Unpaired and Naïve conditions. To construct the maps, XY coordinates for each pMAPK activated cell were categorized into bin that measured 50 µm2. The data points that fell into each bin were counted and placed into a matrix that fit the anatomical dimensions of the LAd (1200 µm2). The colors for each bin reflect an estimation of spatial density from low (blue) to high (red). (TIF) [file pone.0015698.s003.tif]
