# Supplementary material for: Pavlovian Fear Conditioning Activates a Common Pattern of Neurons in the Lateral Amygdala of Individual Brains
Source: PLoS One. 2011 Jan 12;6(1):e15698. doi: 10.1371/journal.pone.0015698 (PMC3020219; doi:10.1371/journal.pone.0015698)
Supplement: Results S1 — A topography of amygdala neurons. (DOCX) [file pone.0015698.s004.docx]

**Supplementary Results**

**Behavior.** In the parallel behavioral test group, animals in the P5 and UP5 groups displayed equivalently high freezing to context compared to the N group when placed back into the original training environment 24 hrs following training for the conditioned fear memory. Three days following the contextual fear test, animals in the P5 group showed enhanced levels of freezing relative to the UP5 (*p* = .00004) and N (*p* = .00001) conditions when the CS was presented in a novel environment (Figure 2). These results indicate that the fear conditioning protocol employed was sufficient to induce a long term fear memory specific to the association of the tone and shock.

**pMAPK neuron density**. Multivariate ANOVA (MANOVA) was used to detect group difference on LA subnucei. The assumption of equality of covariance was checked using Box’s test. A significant interaction was followed by Bonferroni corrected one-way ANOVA’s on individual LA subnuclei. A subsequent Bonferroni post hoc test was carried out on the individual LA subnuclei to compare across conditions. Multivariate ANOVA revealed an interaction of LAd, LAvm and LAvl subnuclei (F_6, 18_ = 4.5; *p* = .006). Follow-up one-way ANOVA revealed significant differences between conditions that were restricted to the LaD (F_2,10_ = 11.5; *p* =.003). Subsequent post hoc comparison within the LAd showed a greater density of pMAPK activated neurons in the P5 relative to the UP5 (*p*=.03) and N (*p*= .002) groups (Figure 2).

**Spatial principal components analysis.** Seven components, accounting for 90.7% of the variance in the data set, were extracted in the sPCA. Topographical grid maps were created using a color-scale reflecting the components loading values for each bin. These maps depict the distribution of factor loadings for components 1-7 (Figure 3). The spatial pattern of loading values for component 1 best represented the difference in spatial distribution of pMAPK labeled neurons between the P5 and the two control conditions (Figure 3). Component 1 accounted for 22.3% of the total variance for the data set. ANOVA revealed a significant difference for the factor scores of the experimental conditions for component 1 (F_2,10_ = 11.1; *p* = .003). This result confirms that the spatial distribution of pMAPK labeled neurons associated with component 1 was related to the experimental manipulation. The score for the P5 group was greater than either the UP5 (*p*=.007) and N (*p*=.003) groups, suggesting a spatial pattern of pMAPK labeled cells that was unique to P5 conditioning. Importantly, subsequent comparison of group means for the remaining factor scores (ANOVA with Bonferroni correction) did not reveal significant differences with respect to condition. This result supports the finding that component 1 represents a unique pattern of variance that is related to Pavlovian auditory fear conditioning (Figure 3-4).

**Multiple comparisons analysis.** Multiple comparisons of the number of cells in the individual bin between groups were tested using one-way ANOVA. For the problem of inflated Type II error for multiple comparisons, α was set at α/n for each comparison. All mean values throughout the text are expressed as a mean ± the standard error of the mean. *p* ≤ 0.05 was considered statistically significant for all statistical comparisons except multiple comparisons, in which case *p* ≤ 0.002 was considered significant. The total number of pMAPK activated cells within each bin was compared across groups. Results showed a significantly greater number of pMAPK cells in the P5 group for bin 7 (*p* = .001) and 11 (*p* = .0001) relative to the UP5 group. Similarly, more pMAPK cells were found in the P5 relative to N group for bin 7 (*p* = .002) and 11 (*p* = .002). In addition, bin 13 was found to posses more pMAPK labeled cells in the P5 compared to N group, although this result only approached statistical significance (*p* = .003). (See Figure 3 for the location of bins 7, 11 and 13). Overall, the P5 group was found to have significantly greater pMAPK activation in bin 7 and 11 compared to controls. The greater number of pMAPK activated neurons in bin 13 for the P5 group most likely contributed to the higher loading value for bin 13 in component 1.

**MePD analysis.** In the MePD, there was no difference in the density of pMAPK labeling (between P5 (109.0 ± 18.0), UP5 (109.7 ± 23.4) and N (91.2 ± 27.7) conditions. These data are in accord with others, indicating that the medial amygdala does not play a role in acquisition of fear memory [1]. sPCA extracted a total five components that accounted for 56.3% of the variance. Component scores did not differ between experimental groups indicating that the variability in the spatial distribution of pMAPK labeling in the MePD was not dependent on the experimental manipulation.

REFERENCES

1. Nader K, Majidishad P, Amorapanth P, LeDoux JE (2001) Damage to the lateral and central, but not other, amygdaloid nuclei prevents the acquisition of auditory fear conditioning. Learn Mem 8: 156-163.

2. Paxinos G, Watson C (2007) The rat brain in stereotaxic coordinates. London: Elsevier Academic Press.
